# Long march for healthy research ecology

**DOI:** 10.1093/nsr/nwz126

**Published:** 2019-09-25

**Authors:** Wei Yang

**Affiliations:** Professor of Zhejiang University and Former President of the National Natural Science Foundation of China

In recent years, the research community in China has launched five campaigns against research misconduct. The first one started at the turn of this century and enabled whistleblowers to flag fabrication, falsification and plagiarism violations during promotions and in talent reviews. Investigations would proceed when the allegation contained verifiable details. Clear signals were sent to researchers, who took the chance of ‘not getting caught’. The second campaign started around 2005 and sought to eliminate duplicate submissions for publication in a different language. In 2007, the Chinese Medical Association established the rule of no duplicate publication in a different language. The copyright law in China also was revised. The third campaign involved the engagement of similarity checks for dissertations, grants and journal submissions. Universities began to perform a full check on all theses submitted for degrees. The National Natural Science Foundation of China installed similarity check software in their system for all submitted grant proposals. All similarities were classified according to their severity and earmarked during the subsequent peer reviews.

Since 2006, the retractions from China have grown rapidly. The reports by *Science* and *Nature* on the misconduct of a young associate professor from Zhejiang University, Haibo He, triggered the fourth campaign against research misconduct. The China Association for Science and Technology and the Ministry of Education of the People's Republic of China initiated massive training programs for research newcomers. At the start of each academic year, the universities and research institutes provide mandatory training programs on research integrity for their new graduate students and faculty members. In the time period of 2012–2013, large-scale retractions of medical papers from China appeared in publications of major publishers, including Springer and Elsevier. The new phenomena of ghostwriting and ghost-reviewing were revealed. These retractions tamed the reputation of Chinese researchers. Consequently, the fifth campaign against misconduct was launched after those high-profile retractions cases featured thorough investigations and severe punitive actions. A coalition task force of government agencies also was assembled to launch a full-scale investigation.

Through those campaigns against research misconduct, the ecosystem in Chinese academia has experienced a pivotal change. Based on data released via the Retraction Watch website, the number of retracted papers from mainland China (referring to the papers published in that year and retracted later) in the year of 2010 was 4117 among 5040 world-wide retractions. The retractions from China, the world-wide retractions, as well as the China-share in the world-wide retractions (about 82%) all reached their historical heights in 2010. Such retraction peaks also occurred for Japan around 1998 and for Germany in the early 1990s. Thanks to the combinational efforts of the entire research community, the number of retracted papers published in 2018 from mainland China substantially declined to 76 (among 268 world-wide retractions).

Two issues in research ecology remain: research ethics and academic lobbying. The gene-editing of human embryos by Jiankui He of Southern University of Science and Technology caused global criticism. The ethics guidance is a more intricate issue than research integrity, and the difficulties in China are compounded by the following aspects. The ecosystem in China is geared more towards black-and-white regulation, but we now are confronted with many gray areas of ethical uncertainty. Most ethical issues are highly professional and cover broad research areas. The ethical rulings evolve with time, while many ethical judgements in China still are tied to traditional values. Violations of the ethical codes frequently are covered by colorful jackets, such as ‘searching new cures of deadly diseases’ along with the impulse for ‘disruptive innovations’. China is relatively unfamiliar to modern ethical research and only has a couple of research journals in ethics.

The other difficult issue is academic lobbying. During the evaluations for research proposals and talent reviews, phone calls, e-mails and text messages flow to the (potential or actual) reviewers for favors from different angles. This lobbying not only clouds academic judgement, but also corrupts the research ecology as winning the competition often depends on personal connections. Worse still, it erodes the trust of scientists in the academic community, media and administrations. To apply stringent regulation to these evaluation procedures also leads to the dilemma of treating the experts as potential offenders. The key is to foster the academic honor of the reviewers and to set up a chain of regulatory mechanisms responsible for ensuring reliability at all links leading to the final evaluation decision.

